# Towards REPO_4_ nanocrystal-doped optical fibers for distributed sensing applications

**DOI:** 10.1038/s41598-023-40161-1

**Published:** 2023-08-09

**Authors:** V. Fuertes, N. Grégoire, P. Labranche, S. Gagnon, S. LaRochelle, Y. Messaddeq

**Affiliations:** Centre d’optique, Photonique et Laser, Université Laval, 2375 Rue de la Terrasse, Québec, QC G1V 0A6 Canada

**Keywords:** Nanoparticles, Glasses, Nanoparticles, Fibre optics and optical communications

## Abstract

Rayleigh scattering enhanced nanoparticle-doped optical fibers, for distributed sensing applications, is a new technology that offers unique advantages to optical fiber community. However, the existing fabrication technology, based on in situ grown alkaline earth nanoparticles, is restricted to few compositions and exhibit a great dependence on many experimental conditions. Moreover, there is still several uncertainties about the effect of drawing process on the nanoparticle characteristics and its influence on the scattering enhancement and the induced optical loss. In this work, we shed light on all these issues that prevent the progress in the field and demonstrate the suitability of doping optical fibers with YPO_4_ nanocrystals for developing tunable Rayleigh scattering enhanced nanoparticle-doped optical fibers. An exhaustive 3D microstructural study reveals that their features are closely linked to the fiber drawing process, which allow the size and shape engineering at the nanoscale. In particular, the YPO_4_ nanocrystals preserve their features to a large extent when the optical fibers are drawn below 1950 °C, which allows obtaining homogeneous nanocrystal features and optical performance. Fabricated fibers exhibit a tunable enhanced backscattering in the range of 15.3–54.3 dB, with respect to a SMF-28 fiber, and two-way optical losses in the range 0.3–160.7 dB/m, revealed by Optical Backscatter Reflectometry (OBR) measurements. This allows sensing lengths from 0.3 m up to more than 58 m. The present work suggests a bright future of YPO_4_ nanocrystals for distributed sensing field and open a new gate towards the incorporation of other rare-earth orthophosphate (REPO_4_) nanocrystals with pre-defined characteristics that will overcome the limitations of the current in situ grown alkaline earth-based technology.

## Introduction

Nanoparticle-doped silica optical fibers is an emerging field that is attracting an increasing attention in recent years among the scientific community. This is justified by the fact that this technology allows maintaining the several advantages that silica optical fibers offer along with novel functionalities introduced by the nanoparticles incorporated^1–3^. However, factors derived from the presence of those scattering centers, such as the Rayleigh scattering induced, which is intimately related to their features, can limit their applicability. As a matter of fact, the typical extreme temperatures of the manufacturing process, generally above 2000 °C, strongly determine the characteristics of the nanoparticles, which can be modified regarding the initial incorporated ones^4^. Thus, it is required a high control of the different stages involved in the fabrication process to be able of maintaining the functionalities pursued in the engineered-silica optical fibers.

One of the current trends in nanoparticle-doped optical fibers is to enhance the Rayleigh scattering, while controlling the induced optical fiber attenuation. The detection of the intrinsic Rayleigh scattering along the fiber is used in distributed optical fiber sensors (DOFS) as the spatial signature of the fiber, which is sensitive to parameters such as strain, temperature or refractive index, among others, with high spatial resolution along the entire fiber under test^5,6^. In particular, optical backscatter reflectometry (OBR) is one of the most popular methods that exploits Optical Frequency Domain Reflectometry (OFDR) to measure the Rayleigh scattering in the optical fiber by means of the backscattered light^7^. The growing interest of this method in last years, suitable for sensing lengths below 100 m, is explained by its high sensitivity combined with a spatial resolution that can reach the sub-millimeter scale^8,9^.

Recently, it was demonstrated that Rayleigh scattering enhanced nanoparticle-doped optical fibers are highly promising for distributed sensing applications since they present several advantages with respect to other methods considered in literature^8,10–12^. Apart from the fact that a better trade-off between scattering enhancement and optical losses have been demonstrated^13–15^, these nanoparticle-doped optical fibers can be manipulated as standard fibers, thereby facilitating their applicability. This approach was firstly demonstrated for MgO-based nanoparticle-doped fibers, co-doped with erbium, which were suitable for distributed sensing of refractive index, strain, temperature and 3D shape sensing^16^. In this type of fiber, MgO-based nanoparticles are grown in situ in the core of preforms and fibers with a random size and a random distribution pattern. However, the strong dependence of the Rayleigh scattering enhancement and the optical attenuation on the random size of nanoparticles and their random distribution^17^, hinders to some extent this approach in terms of reproducibility and scalability. Additionally, although MgO-based nanoparticles were optimized up to 48.9 dB and 14.3 dB/m, respectively, for Rayleigh scattering enhancement and two-way attenuation^18^, sensing lengths were still restricted to less than 3 m. In all these works, solution doping approach was used for the preform preparation, with precursor solution concentrations of 0.1 M of MgCl_2_. Since no phase separation phenomenon was observed for lower concentration values than 0.1 M, a large density of phase-separated nanoparticles was generated, which consequently increased the optical attenuation of the optical fibers. We overcame that problem^13^ and showed that the slight modification of the silica-based matrix, with certain amount of phosphorus and germanium, allows the formation of phase-separated Ca-based nanoparticles at soaking solution concentrations of CaCl_2_ as low as 0.005 M. Consequently, the designed Rayleigh scattering enhanced Ca-based nanoparticle-doped optical fibers were suitable for long-range sensing lengths, from 5 m to more than 200 m, with a tunable enhanced backscattering in the range 25.9–44.9 dB along with relatively low two-way optical losses, 0.1–8.7 dB/m. Nevertheless, in that work, the Ca-based nanoparticles were also grown in situ, and the phase separation phenomenon, and therefore the nanoparticle features, showed a great dependence on several preform fabrication conditions such as vitrification temperature, soaking solution concentrations and composition of the silica-based glass. Moreover, we suggested that during drawing process the nanoparticles underwent a dissolution and re-nucleation as a function of drawing temperature, which strongly affected their morphology and size. We also found a great experimental dependence for Sr-based and Ba-rich nanoparticles in situ growth^15,19^.

Thus, even though alkaline-earth nanoparticles grown in situ in silica-based optical fibers have shown unquestionable high performance for distributed sensing applications, the development of novel nanoparticle-doped DOFS with a larger control of nanoparticle size, shape or composition as well as less dependence on all experimental conditions, will ease their reproducibility and scalability. Moreover, to date, the abovementioned approach is limited to few compositions (Mg, Ca, Sr, Ba) which restricts the applicability of this technology. The discovery of new suitable compositions that can be ex situ grown and incorporated into the core of optical fibers, with pre-defined features designed according to the targeted purpose and remaining invariant during the manufacturing process, will overcome all abovementioned limitations for in situ grown alkaline-earth nanoparticles and would have a significant impact on potential future distributed sensing applications. However, this remains a challenge, partly due to the several uncertainties that exist about the impact of drawing process on the nanoparticles and its correlation with the scattering induced, since these aspects are hardly considered in literature, so far, not only in the field of distributed sensing but also in the whole field of nanoparticle-doped optical fibers.

Recently, we have demonstrated the fabrication of cubic-shaped and rod-shaped YPO_4_ nanocrystal-doped optical fibers, which to the best of our knowledge was the first time that non-spherical nanoparticles are incorporated into silica-based optical fibers^4^. However, to date, their suitability for distributed sensing has not been assessed. Moreover, the fact that rare-earth orthophosphates present high melting temperatures, up to 1900–2100 °C, not only YPO_4_ but also other compounds such as RE = La, Ce, Pr, Nd, Sm, Er, Gd^20,21^, which along with their high refractive index, make them promising materials to be incorporated as ex situ grown nanoparticles for future DOFS^22^. In fact, it has been recently demonstrated that ex situ grown YbPO_4_ nanocrystals, incorporated into fibers, also survive the fiber drawing process^23^.

In this context, the aim of this work is to discover and deeply study new suitable compositions that allow designing novel tunable Rayleigh scattering enhanced nanoparticle-doped optical fibers to improve and expand the current alkaline earth-based technology. Therefore, the assessment of the suitability of our novel YPO_4_ nanocrystal-doped optical fibers for these applications, as well as a thorough analysis of the impact of the fabrication process, including the variation of the initial solution concentration, on the nanocrystal features and its correlation with the induced Rayleigh scattering and optical losses will provide the basis for future REPO_4_-nanocrystal doped optical fibers for distributed sensing field. For this purpose, OBR measurements are performed and correlated with a microstructural 3D study of the nanocrystals in the fiber core by SEM, FIB-SEM and HRTEM.

## Material and methods

### Preform and fiber fabrication

YPO_4_ nanoparticle-doped preforms were fabricated by the conventional MCVD method along with solution doping technique. Solution doping process was carried from different solutions of ex situ synthesized Y_3_Al_12_O_5_ (YAG) nanoparticles in water, at concentrations of 0.01 M, 0.015 M and 0.025 M. YAG nanoparticles, synthesized by combustion method, act as precursors of YPO_4_ nanocrystals, as we showed in our previous work^4^. The high reactivity of the silica-based glass, doped with ~ 5.5 mol% of germanium oxide and ~ 2.5 mol% of phosphorus oxide, at the elevated temperatures of manufacturing process, favors the disappearance of YAG nanocrystals and consequent nucleation of YPO_4_ nanocrystals in the preform core. The nanoparticle-doped silica soot was vitrified at 1800 °C while preform collapse was above 2000 °C. Fabricated preforms are labeled, hereafter, as preform #1–3, in increasing order of concentration, that is, preform #1 is made from a soaking solution of 0.01 M while preform #3 from the solution 0.025 M. In the range considered, the nanoparticle density is the most noticeable parameter affected, increasing as soaking solution concentration increases, which turns the core from transparent, in preform #1, to slightly milky, in preform #3 (Fig. S1a). The refractive index contrast (Δn) values measured for the preforms are 9 × 10^–3^, 9.1 × 10^–3^ and 9.5 × 10^–3^ for preforms #1, #2 and #3, respectively (Fig. S1b). Therefore, the increase of nanoparticle concentration slightly increases the refractive index contrast of the corresponding preform.

The optical fibers were drawn from preform #1–3 at temperatures of 1900 °C, 1950 °C, and 2000 °C, with a drawing speed of 5 m/min. The external diameter of the optical fibers is around 125 μm while the core diameter is 10 μm, which allows the manipulation as in standard telecom fibers.

### Structural, microstructural characterization and optical characterization

Microstructural characterization of preforms and fiber was carried out by means of a FEI QUANTA 3D FEG Scanning Electron Microscopy (SEM), with a resolution of 1.5 nm at 30 kV in Secondary Electron (SE) and low vacuum mode. Composition of the core of preform and fibers was analyzed by means of Energy Dispersive X-ray (EDX) detector. Focused ion beam (FIB) along with SEM were used to investigate the nanoparticle features in the volume of the fiber. The volume was etched by using a 30 kV and 3 nA of Ga ion beam. SEM micrographs in the FIB-SEM mode were obtained at 52° with respect to the surface of the fiber sample. Particle size along the z-axis was measured taking into consideration the tilt correction. Dimensions of the analyzed area after etching process were around ~ 5 × 1 × 7 µm. Image analysis was performed by ImageJ software. HRTEM images were acquired using a FEI Titan low-base (LB) 80–300 TEM equipped with a CEOS image corrector, resulting in a point resolution of 0.8 Å. The microscope was operated at an acceleration voltage of 300 kV and images acquired using a Gatan CCD.

Regarding optical characterization, refractive index profile of the fabricated preforms was measured by means of a PK2610 Preform Analyzers from PhotonKinetics. OBR measurements of the Rayleigh scattering enhanced nanoparticle-doped optical fibers were carried out by using a commercial Luna OBR 4600, characterized by a sensitivity of − 130 dB and a spatial resolution of 20 μm. For these OBR measurements, the different nanoparticle-doped fibers were spliced to a SMF-28 fiber pigtail ending in a FC/APC connector and connected to the OBR 4600. The fibers were evaluated with a laser input centered at 1550 nm and sweeping in a wavelength range of 43 nm, with a total of 16,384 sensing points per analysis.

## Results and discussion

### Microstructural characterization of nanoparticles on preforms and fibers, in-plane and along drawing direction: SEM, FIB-SEM and HRTEM

While in our previous work^4^ we have focused on the compositional and structural details of the nucleated YPO_4_ nanocrystals on the optical fibers, in this current work, we explore the effect of varying the initial solution concentration on the preform nanoparticle characteristics as well as the effect of fiber drawing temperature. Moreover, FIB-SEM is also used, and it is included in this section, to deepen and investigate the effect of fiber drawing on the microstructural nanocrystal features along drawing direction, which has not been analyzed to date for this compound. Furthermore, the next section presents the nanoparticle features correlation with the optical performance of the developed fibers for their application as distributed optical fiber sensor.

**Figure 1:**
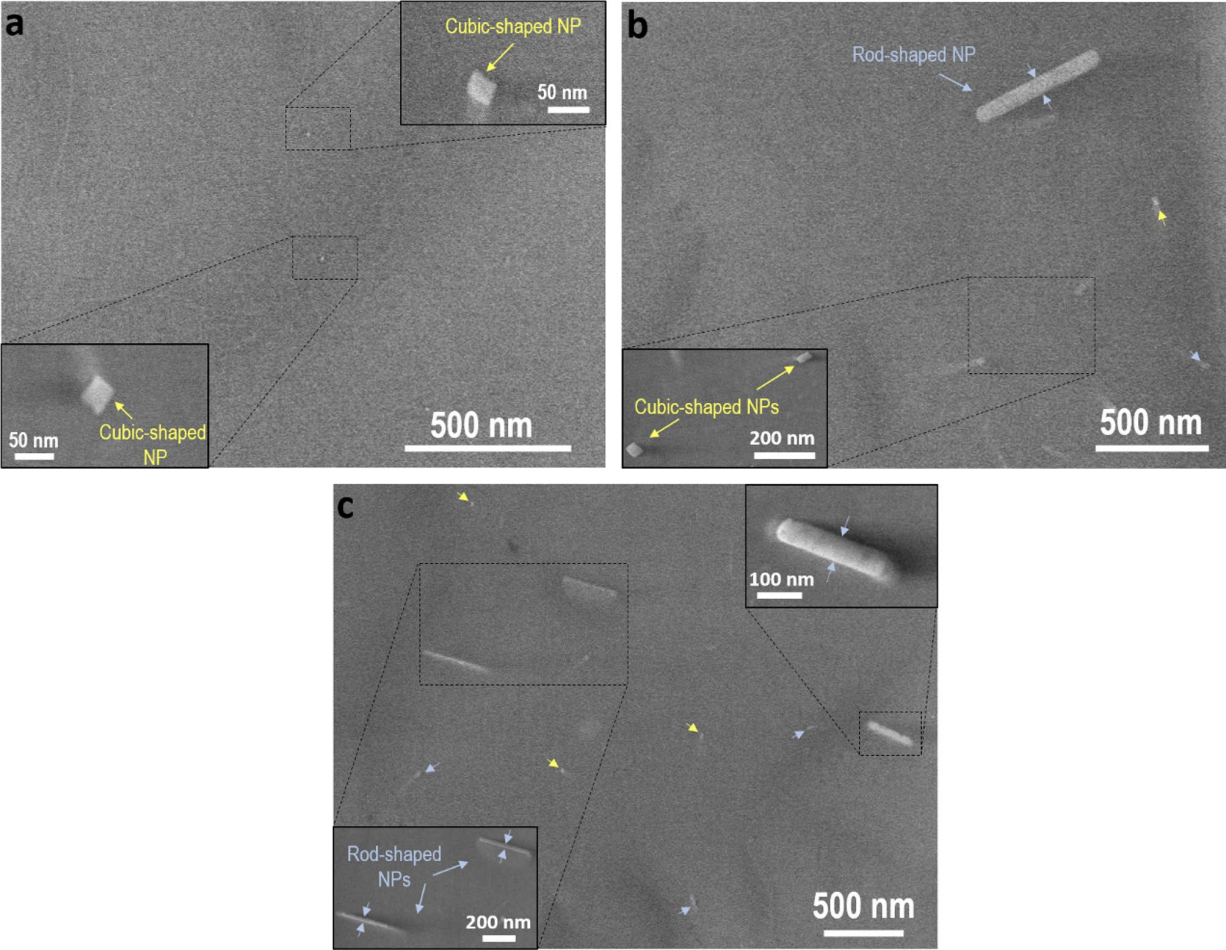
Representative SEM micrographs of the core of: (**a**) preform #1; (**b**) preform #2 and (**c**) preform #3. The insets show an enlargement of the corresponding marked area in which the morphology and size of nanoparticles are displayed in more detail. Rod-shaped and cubic-shaped nanoparticles are marked with light blue and yellow arrows, respectively, in the preform core micrographs.

With the aim of fabricating DOFS with tunable Rayleigh scattering enhancement, solutions with three different concentrations were considered for the fabrication of the preforms during solution doping process, in the range 0.01–0.025 M (see “Material and methods” for further details). The very low nanoparticle density for preform #1,2, caused large transparency of the corresponding preform cores (Fig. S1a), but also hampered an in-depth analysis as the one carried out for preform #3. In contrast, for preform #3, the high nanoparticle density caused a slightly milky appearance for the core but allowed in-depth analysis of the nanocrystal features (Fig. S1a). In the range considered, the nanoparticle density is the most noticeable parameter affected by the soaking solution concentration, increasing as concentration increases, which reduces the transparency. Figure 1 shows representative SEM micrographs of the preform core for the preforms #1–3. Regardless the solution concentration considered, the nanoparticles observed present mainly two morphologies: rod-shaped, and cubic-shaped, and thus their shape is not affected by the solution doping concentration. For preform #3, based on our previous work^4^, rod-shaped nanoparticles constitute around 55% of the total nanoparticles observed while cubic-shaped ones are 45%. These nanostructures correspond to a YPO_4_ composition. Figure 1a shows a representative SEM micrograph of the core of preform #1. This preform presents the lowest concentration considered in this work, 0.01 M, which is translated into the presence of well dispersed nanoparticles with an inter-particle distance of generally several microns. The closest nanoparticles were found at around 400 nm, as it can be seen in Fig. 1a. Figure 1b shows the core of preform #2, in which it is seen that the increase of concentration, from 0.01 to 0.015 M, has a direct impact on the inter-particle distance, which is reduced regarding preform #1, as well as on nanoparticle density, which is increased in the preform core, as expected. The closest nanoparticles found are placed around 300 nm from each other while in preform #1 was ~ 400 nm, although a large inter-particle distance is generally observed in the entire core, above 1 μm. Finally, for preform #3, it can be clearly seen a remarkable increase in nanoparticle density along with a decrease of the minimum inter-particle distance below 300 nm (Fig. 1c). The increase of concentration is also evinced in the presence of particles under the analyzed surface, which is manifested as blurry spots in Fig. 1c. Rod-shaped and cubic-shaped nanoparticles are marked with light blue and yellow white arrows, respectively, to ease their identification (Fig. 1c). Cubic-shaped nanoparticle distribution size ranges from 23 to 72 nm, and with an average size of roughly 44 nm^4^. The aspect ratio of the rod-shaped nanoparticles is overall comprised in the range of 5–8. Consequently, the core becomes more translucent (Fig. S1a) and the refractive index contrast between core and cladding is subtly enhanced from 9.0 × 10^–3^, in preform #1, up to 9.5 × 10^–3^, in preform #3 (Fig. S1b). It is worth mentioning that no additional annealing is required in this kind of preforms to attain the nanoparticles after the preform fabrication process, as occurs with other oxide nanoparticles considered as dopants in optical fibers^2^. All these features, that is, low nanoparticle size with relatively large inter-particle distance, mainly for preform #1, 2, and distributed homogeneously along the preform core are key to achieve low-loss nanoparticle-doped optical fibers^13^.

**Figure 2:**
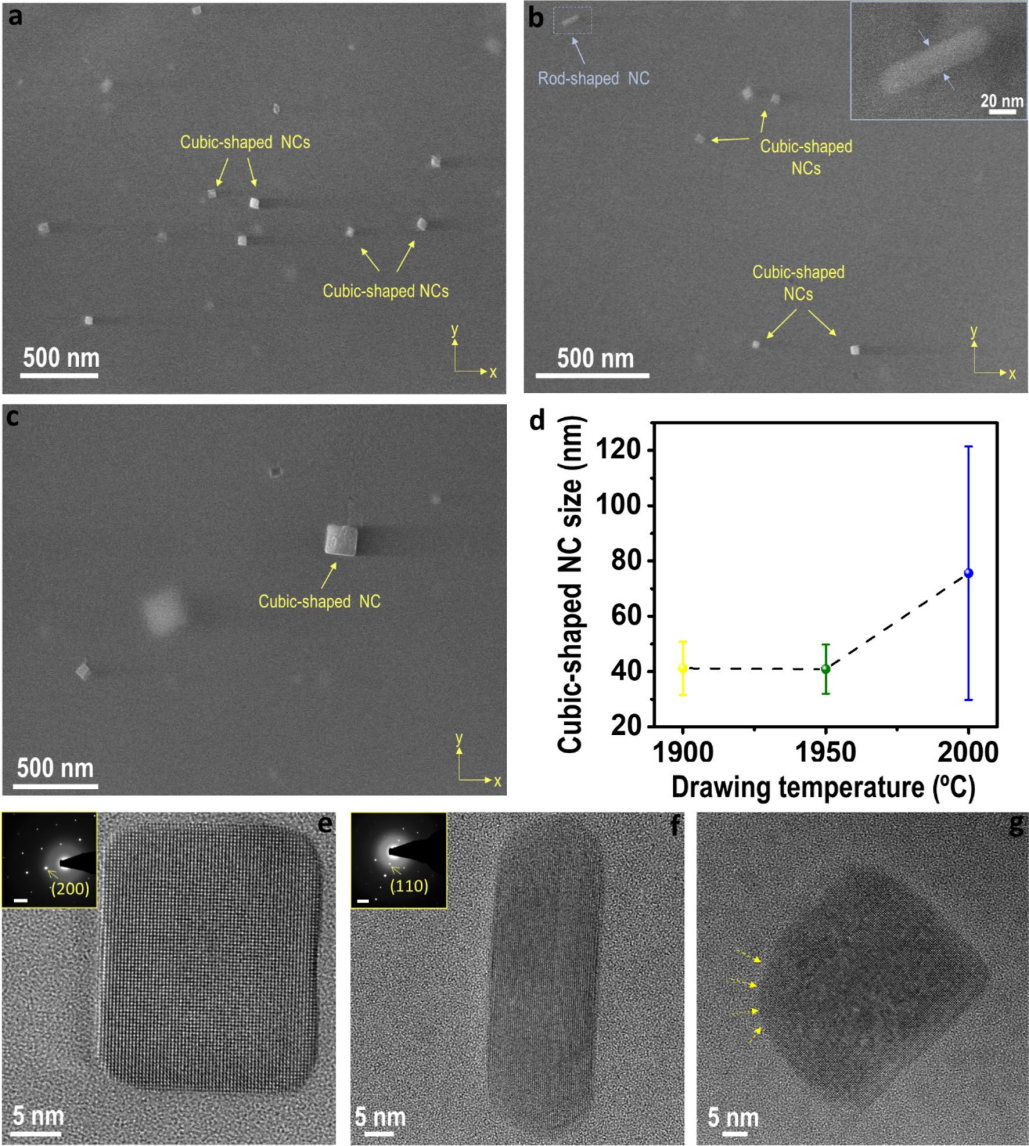
Representative SEM micrographs of cross-sections for the YPO_4_ nanocrystal-doped optical fibers drawn from preform #3 at: (**a**) 1900 °C, (**b**) 1950 °C and (**c**) 2000 °C; (**d**) Mean cubic-shaped nanocrystal size vs drawing temperature, along with the standard deviation (σ) plotted as error bars; HRTEM and the corresponding SAED patterns (scale bars of 2 1/nm) for the three characteristics morphologies: (**e**) cubic-shaped, (**f**) rod-shaped and (**g**) rounded cubic-shaped nanocrystals.

Optical fibers were drawn from the preforms previously analyzed, at three different drawing temperatures, ranging from 1900 to 2000 °C, in order to investigate their impact on the nanoparticle features and allow their engineering, extending the applicability of the corresponding DOFS. Factors such as nanoparticle density, inter-particle distance, particle size or morphology need to be investigated since they will determine the induced Rayleigh scattering of the optical fibers. With the aim of attaining a large and representative statistics about the whole fiber core, the fiber samples with the largest density of nanoparticles were analyzed, that is, fibers drawn from preform #3. Representative in-plane SEM micrographs of cross-sections at the different fiber drawing temperatures considered are shown in Fig. 2a–c. It is seen that as drawing temperature increases, inter-particle distance increases and number density of nanoparticles considerably decreases due to the progressive dissolution of nanoparticles in the silica-based glass during the fiber fabrication process. Nanoparticles are placed at less than 100 nm for drawing temperatures of 1900 °C (Fig. 2a), while at 2000 °C, most of the sections analyzed are only composed by few nanoparticles, isolated in a fiber core of 10 microns, and with the closest ones at around 300 nm from each other. As it is shown below, all nanoparticles are crystalline, and referred as nanocrystals hereafter. Particle size of the predominant nanocrystals, cubic-shaped nanocrystals, is depicted in Fig. 2d as a function of drawing temperature^4^. The error bars indicate the standard deviation (σ). Below 2000 °C, the cubic-shaped nanocrystals size presents high homogeneity, with sizes in the range 20–70 nm, an average size of ~ 41 nm and σ of ~ 10 nm. HRTEM images and the corresponding SAED patterns, included as insets, are depicted in Fig. 2e and f for both the cubic shaped- and rod-shaped nanocrystals, which are identified with tetragonal and monoclinic phase, respectively^4^. Structural differences between these nanocrystals justify the fact that as temperature rises, the rod shaped-nanocrystals are dissolved, and hardly detected, while cubic-shaped ones prevail (Fig. 2a–d), since GdPO_4_ monazite structure, the closest one to YPO_4_ monazite, has a melting point of ~ 1900 °C, lower than xenotime, ~ 2000 °C^20,24^. The reaction with the glass at 2000 °C is revealed in the increase of the size (Fig. 2c,d), with mean values of ~ 76 nm and σ = 46 nm. While the cubic-shaped nanocrystals maintain their sharp edges in the temperature range of 1900 °C ≤ T < 2000 °C, beyond that temperature, the reaction with the silica-based glass of the fiber core start to turn them rounded as shown in the HRTEM micrograph of Fig. 2g, with nanocrystals that reach up to 190 nm (Fig. S2). It is worth remarking that as fiber drawing temperature increases, the smallest nanoparticles tend to disappear while the existing bigger nanoparticles increase their size, which is more noticeable in the range of 1950 < T ≤ 2000 °C^4^. Moreover, there is an overall nanocrystal dissolution. This shape control is also verified for lower nanoparticle solution doping concentrations, as it can be seen for instance in the SEM micrograph included in Fig. S3 for an optical fiber drawn at 1900 °C from preform #1.

**Figure 3:**
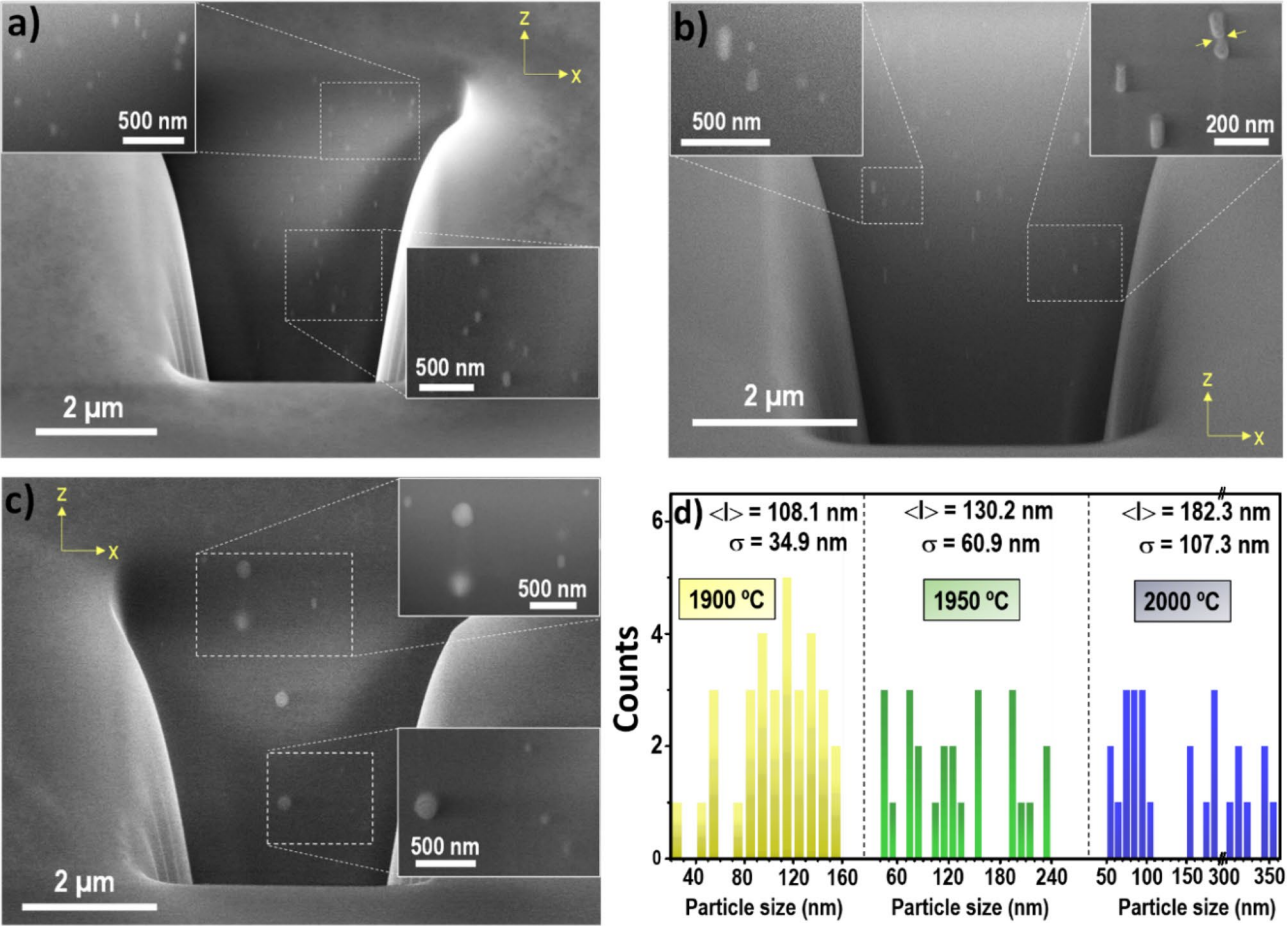
FIB-SEM micrographs of transverse sections from the optical fiber core for fibers drawn from preform #3 at: (**a**) 1900 °C, (**b**) 1950 °C and (**c**) 2000 °C. Insets show in more detail the influence of drawing temperatures on the nanoparticle features in the fiber drawing direction. (**d**) Histograms depict the mean particle size along drawing direction (< l >), standard deviation (σ) and particle distribution sizes for the nanocrystals at the corresponding drawing temperature.

It is worth mentioning that for this new kind of nanocrystal-doped optical fibers there are plenty of questions to answer regarding the nanocrystals in the fiber core volume such as their shape and size. Therefore, with the aim of deepening more into the influence of drawing process and correlate with the in-plane SEM observations (Fig. 2) and the backscattering enhancement later, a FIB-SEM analysis was carried out for the optical fibers drawn from preform #3. Figure 3 displays SEM micrographs of transverse sections and show that the nanocrystals in the fiber core tend to be elongated along the drawing direction, for temperatures below 2000 °C, as a direct consequence of fiber drawing process. The mean aspect ratio for the nanocrystals changes from 2.1 to 2.7, in the drawing temperature range of 1900–1950 °C and decreases to 1.6 at 2000 °C. Density of particles is reduced as drawing temperature increases, which increases consequently the inter-particle distance, as it was also stated from the in-plane observations (Fig. 2a–c).

Based on the previous discussion, these observed nanocrystals in the fibers drawn below 2000 °C (Fig. 3a,b) should mostly correspond to the YPO_4_ cubic-shaped ones observed in Fig. 2, since they comprise the majority, ~ 70%^4^. The particle distribution size of the nanocrystals in the fiber core along the drawing axis tends to broaden as temperature increases, while the average size is enlarged, as shown the histograms depicted in Fig. 3d. At 1900 °C, nanocrystals between 23 and 151 nm in length are found, with an average length of around 108 nm. Taking into consideration the average size of the nanocrystals observed in the XY plane (Fig. 2d), it can be concluded that the nanocrystals increase their mean size in the drawing axis, in a factor of ~ 2.7. That increase in Z-axis is more pronounced as the temperature increases. The mean length increases to 130 nm for a drawing temperature of 1950 ºC, and 182 nm at 2000 °C. At this temperature of 2000 °C, nanocrystals between 57 and 352 nm are measured, with a more spherical shape, which confirms that the presence of the smallest nanocrystals disappear as temperature increases, instead, the largest ones grow even more. Moreover, as it is seen in the right-top inset of Fig. 3b, some particles show the formation of bottlenecks between them. These observations may suggest that a coalescence mechanism can be involved in the nanoparticle growth, although an Ostwald ripening was previously suggested^4^. Further studies are needed to confirm the exact mechanism involved. All in all, the smallest nanocrystals tend to disappear as temperature increases while the existing larger ones increase their size, which causes a standard deviation increase (Figs. 2d, 3d), and the number density of nanocrystals is reduced. The larger spherical shape along with more inhomogeneous particle size distribution at 2000 ºC, with σ = 107 nm, due to the reaction with the silica-based glass, it is consistent with the behavior observed from in-plane SEM analysis.

These observed features along the drawing direction, i.e., the slight size increase along the drawing axis or the formation of bottlenecks, evince the need of investigating the features of nanoparticles in the fiber volume as well, to fully characterize the optical fiber under study, even though in literature it is not usually taken into consideration. All these characteristics as a function of drawing temperature will strongly determine the Rayleigh scattering and optical attenuation of the optical fibers, due to the direct connection between size and density of nanoparticles with these optical properties^13^.

In^25^, it was reported that LaF_3_ nanoparticles incorporated within the core of the fiber, with an initial size of 10 nm, were elongated along the drawing axis and reached sizes up to 300 µm, in length, although some others below 70 nm were also observed, due to some break-up phenomena that occurred during the drawing process. Consequently, a very wide particle distribution size was attained. Furthermore, in our previous work for Ca-based nanoparticles^13^, we suggested that the nanoparticles presented in the preform core, with a mean diameter of 50 nm, were dissolved and re-nucleated throughout the drawing process since their shape was modified, from spherical to elongated, and their size grew up to 500 nm. This extreme behavior is not shown by the YPO_4_ nanocrystals during the drawing step, as these nanocrystals maintain the initial size and shape when lower drawing temperatures are used.

As mentioned, the difficulty of controlling the features of in situ grown alkaline earth nanoparticles, from preform to fiber, strongly determine the possible tailoring of Rayleigh scattering and optical losses in this kind of distributed sensing systems, which is simultaneously restricted to compositions such as Mg-, Ca-, Sr-, and Ba-based. Thus, by incorporating YPO_4_ nanocrystals and properly controlling the fiber fabrication process, some of the limitations related to the use of alkaline earth nanoparticles are overcome, such as the dissolution and re-nucleation with different morphology and size observed during fiber drawing for Ca-based nanoparticles^13^, or the considerable size modification for Sr- and Ba-rich nanoparticles^15,19^.

### Tuning of Rayleigh scattering enhancement and optical losses in YPO_4_ nanocrystal-doped optical fibers: OBR

**Figure 4:**
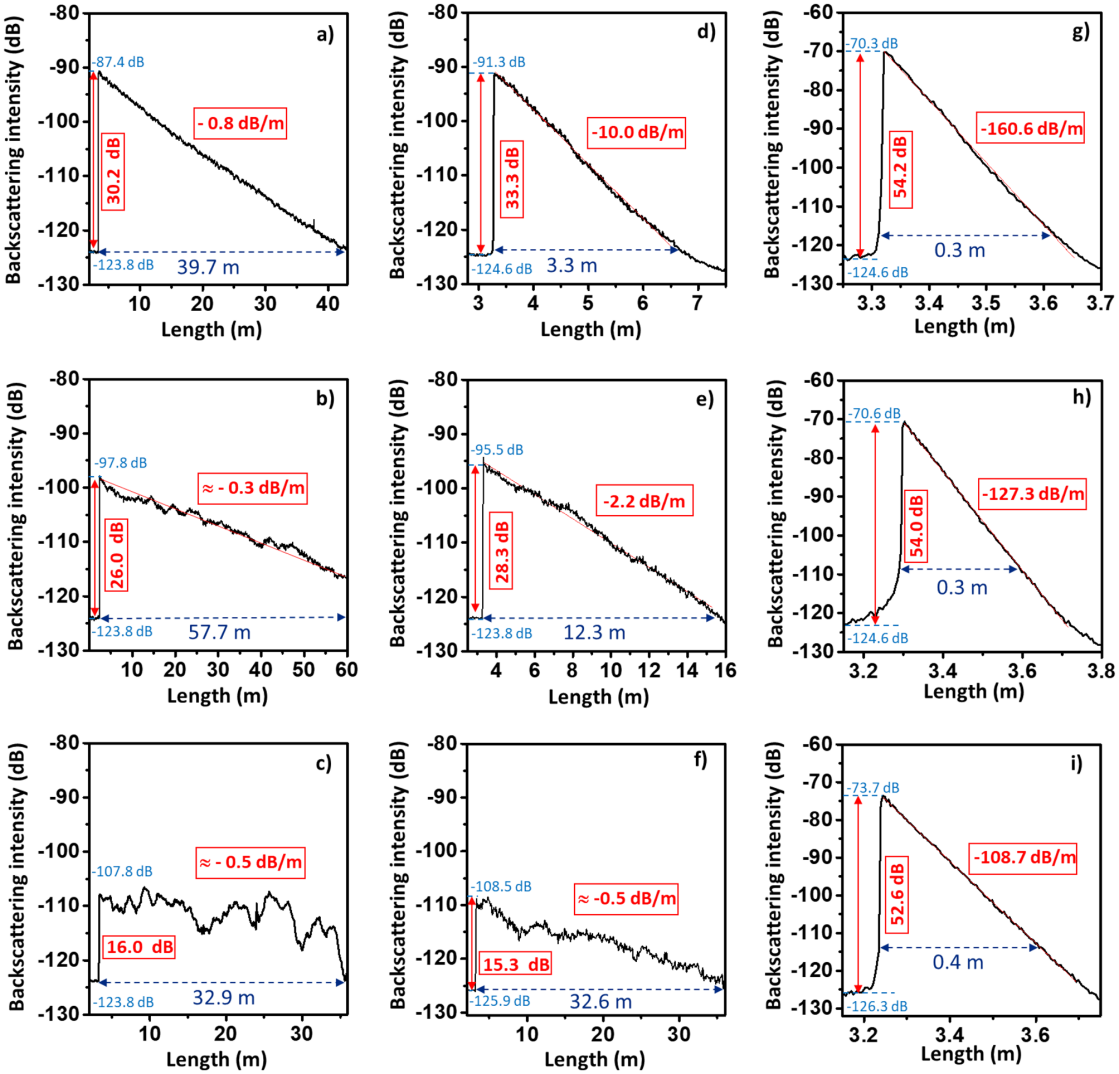
Backscattering intensity *vs* optical fiber length for YPO_4_ nanocrystal-doped optical fibers drawn at temperatures of 1900 °C, 1950 °C and 2000 °C, respectively, from: (**a–c**) preform #1; (**d–f**) preform #2 and (**g–i**) preform #3. The relative enhanced Rayleigh backscattered intensity, with respect to a SMF-28, is indicated with a red arrow in each plot.

To assess the impact of the nanocrystal features on the Rayleigh scattering induced in the optical fibers, the enhanced Rayleigh backscattering intensity has been measured along the fiber length for the different fibers fabricated in this work, that is, from preform #1–3 at drawing temperatures of 1900–2000 °C (Fig. 4). For the measurements, the different fibers were spliced to a SMF-28 fiber, which is considered as reference.

Thus, the enhancement of Rayleigh backscattering intensity for each sample, indicated with a red arrow in Fig. 4, is attained from the difference between the measured intensity before and after the splice point. According to Fig. 4, a tunable enhanced Rayleigh backscattering intensity is obtained, comprised between 15.3 and 54.3 dB, by the control of both drawing temperature and doping concentration. This enhancement of Rayleigh scattering is caused by the presence of the nanocrystals in the core of the fiber, which induce simultaneously an optical attenuation. These optical loss values are estimated from the plots. In the graphs presenting a well-defined linear slope, a linear regression is used (marked by a red line in Fig. 4). On the other hand, for the cases in which larger oscillations are registered in the drop of backscattering signal measurement (Fig. 4c,f), the estimation is carried out considering the drop of the signal from the maximum value to the noise floor, as well as the length that the light travels inside the fiber before reaching the noise floor intensity. Mean estimated values range from 0.3 to 160.6 dB/m (taking into consideration forward and backward propagation loss) and depend on drawing temperature and doping concentration, as it might be observed in Fig. 4.

**Figure 5:**
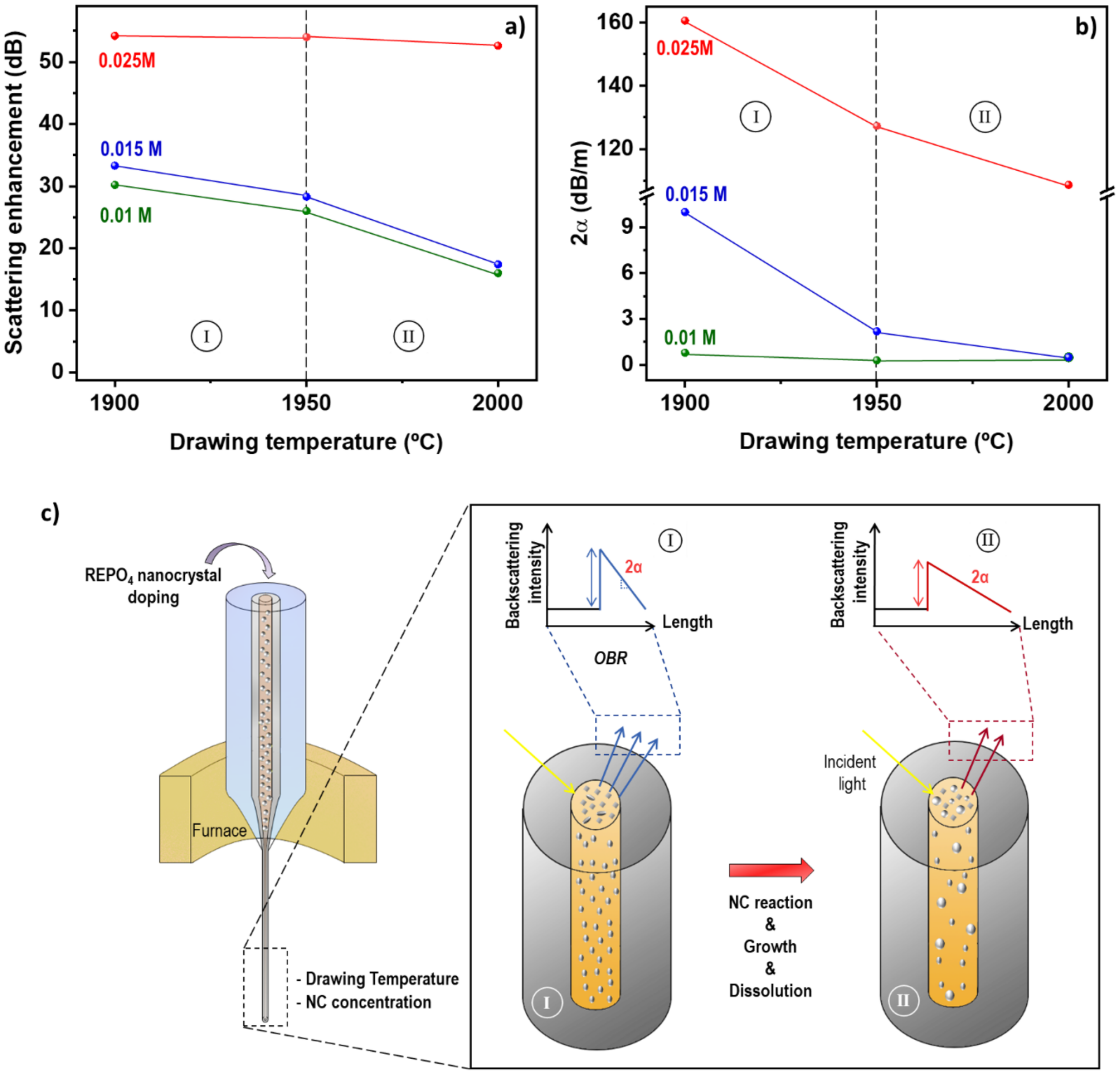
(**a**) Scattering enhancement and (**b**) two-way attenuation, 2α, *versus* drawing temperature for YPO_4_ nanocrystal-doped silica-based optical fibers for the different solution concentrations considered in this work. (**c**) Schematic model that correlates the features of the nanocrystals inside the fiber core, for a given solution concentration, with the Rayleigh scattering enhancement and optical attenuation plotted in (**a**) and (**b**), as a function of drawing temperature. Inhomogeneous scattering appears above 2000 ºC due to the impact of temperature on the initial nanocrystal features.

Overall, at drawing temperatures in the interval 1900–1950 °C and soaking concentrations of 0.010–0.015 M, the Rayleigh backscattering pattern display a sawtooth shape, which start presenting some irregularities as temperature increases. This fact is due to the partial dissolution of the nanocrystals as a function of temperature, which causes inhomogeneous nanocrystal features in terms of morphology, composition (and therefore differences of refractive index between the particles), and particle distribution size along the drawing direction, as discussed in detail in Section “Microstructural characterization of nanoparticles on preforms and fibers, in-plane and along drawing direction: SEM, FIB-SEM and HRTEM”. Consequently, the measured backscattering presents this irregular behavior (Figs. 4, 5a,b). As consequence of these dissolution phenomena less nanocrystals are present, and the backscattering level is reduced as the drawing temperature increases and thus optical attenuation, even though particle size increases (Figs. 2d, 3d). In this temperature interval, for optical fibers drawn from preform #1, Rayleigh backscattering intensity enhancement is reduced around 4 dB, from ~ 30 to 26 dB, while the two-way attenuation undergoes a less significant change, from − 0.8 to − 0.3 dB/m (Figs. 4a,b, 5a,b). In the case of optical fibers drawn from preform #2, the scattering intensity is higher than in preform #1, because of the larger initial nanoparticle concentration, and decreases following a similar rate (Figs. 4d,e, 5a,b), from 33.3 to 28.3 dB. However, optical losses are significantly more reduced, by a factor of ~ 5, from − 10.0 to − 2.2 dB/m. At 2000 °C (Figs. 4c,f, 5a,b), in the concentration interval 0.010–0.015 M, backscattering measurement displays several pronounced oscillations caused by the large inhomogeneities in the size, shape and composition of nanoparticles in the fiber core, as previously discussed. Consequently, tunable long-sensing distances, between ~ 3 m and more than ~ 58 m are attained. In contrast, for preform #3, these backscattering irregularities are less noticeable; however, it is worth pointing out the differences in the scale of x-axis, since the signal vanishes in a much shorter fiber length, < 0.4 m, due to the larger attenuation caused by the larger nanoparticle concentration, which might hide the wavy pattern. The correlation of the backscattering enhancement and the two-way attenuation *versus* the drawing temperature is plotted in Fig. 5a,b, along with a schematic model plotted that consider the nanoparticle features and the correlation with the optical performance of the fibers (Fig. 5c), based on the previous discussion. As previously mentioned, it is anticipated that these findings can be extrapolated to other REPO_4_ compounds which strengthen the impact of these findings.

The good performance observed, in terms of trade-off between scattering enhancement and optical losses, allows sensing lengths from ~ 3 m to more than ~ 58 m, is partly possible because of the suitability of YPO_4_ in terms of Rayleigh scattering cross-section (*σ*_*Rayleigh*_). A simple comparison with other compositions reported for distributed sensing might be carried out, without considering differences in morphology. For instance, for spherical nanoparticles, *σ*_*Rayleigh*_ exhibits a dependency on the diameter of nanoparticles by a power of six and a dependency with the refractive index of the nanoparticles of a power of four^26^. Refractive index of YPO_4_ is 1.72–1.82^27^, while the refractive index of other compositions reported for this application are ~ 1.58 for MgO-based nanoparticles^28^ and ~ 1.70 for CaO-based nanoparticles^13^. The increase of *σ*_*Rayleigh*_ consequently increases the optical attenuation of the fiber, however the linear dependence with the particle density makes it an important parameter to be considered. In fact, the lower *σ*_*Rayleigh*_ for MgO-based nanoparticles, for a given particle size, made it that the considered nanoparticle concentration had to be considerably increased, which is translated into high scattering values in the fiber, but also high two-way optical losses, of tens of dBs/m^18^.

From the results discussed above, it can be established that to attain a good trade-off between scattering enhancement and optical losses that allows long-range sensing lengths, it is necessary to have well dispersed and isolated nanocrystals, generally separated up to several microns. On the other hand, this study reflects that the incorporation of nanoparticles in optical fibers, and in particular for distributed sensing is not a trivial event nor easily controllable, since many factors are involved during fabrication process such partial dissolution of nanoparticles, reaction with the glass, possible inhomogeneities caused during soaking solution process, compositional changes etc., that may lead to not having a linear response in terms of Rayleigh scattering and optical attenuation with solution concentration (Fig. 5a,b). Nevertheless, the behavior measured, and trend observed in all the OBR measurements evince that it is possible to achieve a high control of all these parameters by controlling all the stages of preform and fiber fabrication process.

In the literature, so far, the studies reported about nanoparticle-doped optical fibers for distributed sensing applications were based on alkaline-earth based nanoparticles, grown in situ in silica-based optical fibers, as previously mentioned. However, that approach exhibits large dependence on multiple fabrication factors, and the fiber fabrication process strongly affects to the nanoparticle characteristics. In this present study, we have demonstrated that tetragonal YPO_4_ nanocrystals are hardly affected when the optical fibers are drawn at 1900 °C which allows obtaining homogeneous nanocrystal features and optical performance therefore (Fig. 4a,d,g). The studied drawing temperatures establish the limits for the fabrication of these DOFS with an optimal performance. Further studies in this range of nanocrystal concentration and temperature can allow adapting the targeted features of nanocrystals and therefore their sensing performance for the aimed application. Furthermore, it is reported in literature the possibility of synthetizing YPO_4_ nanostructures with different structures and morphologies such as prisms, sheets, spindles or spheres, apart from cubes and rods^29,30^. Moreover, it is worth noticing that YPO_4_ belongs to the family of RE orthophosphates, which are characterized by very high melting points ~ 1900–2100 °C^20,21^. This suggest that the direct incorporation of any REPO_4_ nanostructure into the glass matrix will preserve the REPO_4_ nanocrystals. Thus, these achievements open up the incorporation of ex situ grown YPO_4_ nanocrystals as well as other REPO_4_ compounds, with pre-defined characteristics in the core. This will allow tailoring and tuning the Rayleigh scattering enhancement of the fibers by means of their shape, composition, and structural features, which will have a significant impact on potential future distributed sensing applications.

## Conclusions

Our work demonstrates the suitability of doping silica-based optical fibers with YPO_4_ nanocrystals for the development of tunable long-range Rayleigh scattering enhanced nanoparticle-doped optical fibers. Moreover, the impact of fiber fabrication process on the microstructural features of the nanocrystals is thoroughly assessed and correlated with the enhancement of Rayleigh backscattering and the induced optical attenuation in the fibers. This is carried out for the first time for a compound that belongs to the REPO_4_ family. Overall, it is observed that tetragonal YPO_4_ cubic-shaped nanocrystals are hardly affected in-plane by drawing process, below 2000 °C, although undergo a slight elongation along the drawing direction. These more homogeneous nanocrystal features are translated into a Rayleigh backscattering enhancement without noticeable irregularities. However, the increase of drawing temperature, to 2000 °C, favors the reaction of the tetragonal YPO_4_ cubic-shaped nanocrystals with the silica-based glass of the fiber core and cause their growth and the rounding of their edges in both transverse and axial direction. Simultaneously, the inter-particle distance is increased, and the particle distribution size broadened, while the number density is reduced. Consequently, as the drawing temperature increases, these features are translated into a decrease of the induced Rayleigh backscattering enhancement intensity and a more inhomogeneous scattering pattern. By controlling both drawing temperature and doping concentration, a tuning of Rayleigh scattering enhancement, from 15.3 to 54.3 dB, compared to a SMF-28 fiber, along with two-way optical losses in the range of 0.3–160.7 dB/m are demonstrated. Consequently, sensing lengths from 0.3 m up to more than 58 m become possible.

These results illustrate the potential of YPO_4_ nanocrystals for distributed sensing applications and add new knowledge to the emerging field of nanoparticle-doped optical fibers. We establish solid guidelines about the main experimental factors that need to be considered along the fabrication process for the successful incorporation of future ex situ grown YPO_4_ and other REPO_4_ nanocrystals. It is anticipated that this will overcome all limitations of the current in situ growth alkaline earth-based technology and improve the existing distributed sensing technology.

### Supplementary Information

Supplementary Figures.

## Data Availability

The datasets used and/or analysed during the current study available from the corresponding author on reasonable request.
