# Supplementary material for: Towards REPO4 nanocrystal-doped optical fibers for distributed sensing applications
Source: Sci Rep. 2023 Aug 9;13:12891. doi: 10.1038/s41598-023-40161-1 (PMC10412647; doi:10.1038/s41598-023-40161-1)
Supplement: Supplementary file 1 — Supplementary Figures. [file 41598_2023_40161_MOESM1_ESM.docx]

**Supporting information**

Towards REPO_4_ Nanocrystal-doped Optical Fibers for Distributed Sensing Applications

*V. Fuertes**^1^*, N. Grégoire*^1^*, P. Labranche*^1^*, S. Gagnon*^1^*, S. LaRochelle*^1^*, Y. Messaddeq*^1^

^1^ Centre d’optique, Photonique et Laser, 2375 Rue de la Terrasse, Université Laval, Québec, (QC), G1V 0A6, Canada

^2^ Canadian Centre for Electron Microscopy, 1280 Main Street West, Hamilton, (ON), L8S 4M12375 , Canada

*Corresponding Author: [victor.fuertes-de-la-llave.1@ulaval.ca](mailto:victor.fuertes-de-la-llave.1@ulaval.ca)

**S1. Characterization of nanoparticle-doped silica-based preforms**

**
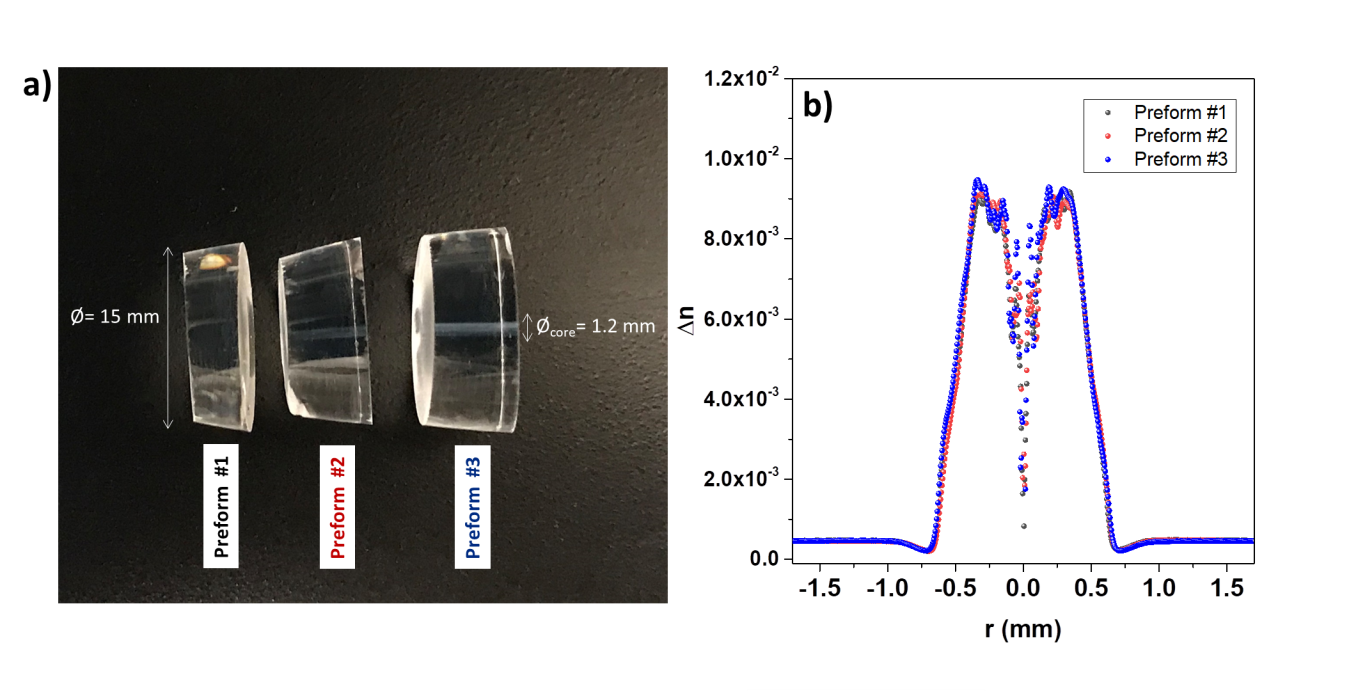
**

**Figure S1**. **a)** Photograph of a section of the fabricated preforms #1-3 that shows a decrease of transparency in the nanoparticles-doped core as soaking concentration increases, that is, from preform #1 to preform #3. **b)** Refractive index profiles of nanoparticle-doped preforms #1-3.

.

**S2. SEM micrograph of** **YPO_4_ nanocrystal-doped silica-based optical fiber drawn at 2000 ºC from preform #3**


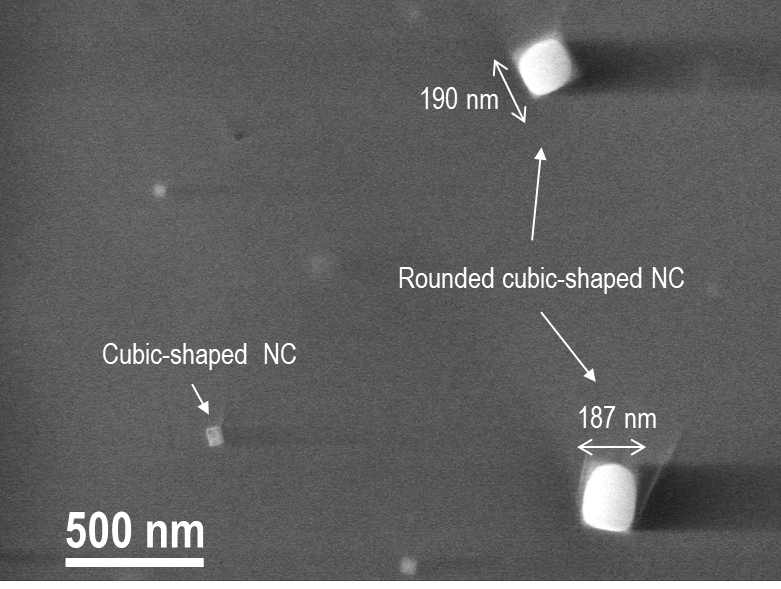


**Figure S2**. **a)** SEM micrograph of the YPO_4_ nanocrystal-doped silica-based optical fiber drawn at 2000 ºC from preform #3 that shows the presence of isolated nanocrystals up to ~190 nm and with rounded edges. This behavior is explained by the considerable reaction between nanocrystals and the silica-based glass of the fiber core that occurs at this drawing temperature.

**S3. SEM micrograph of** **YPO_4_ nanocrystal-doped silica-based optical fiber drawn at 1900 ºC from preform #1**


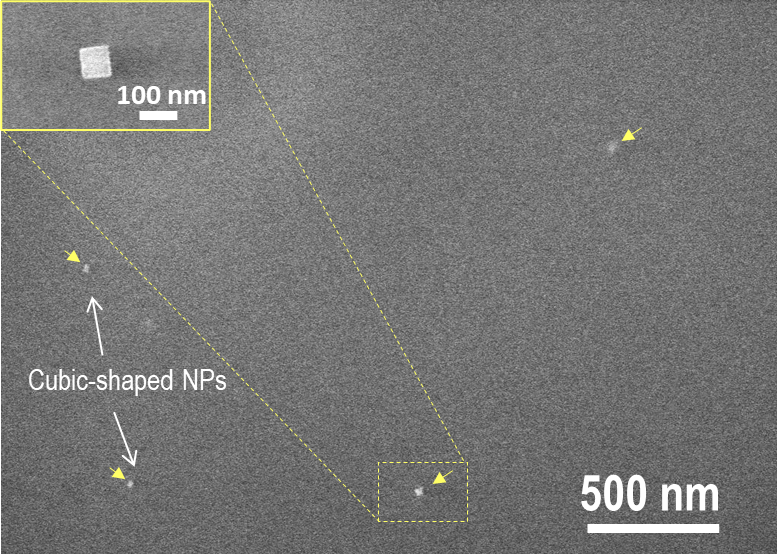


**Figure S3**. SEM micrograph of YPO_4_ nanocrystal-doped silica-based optical fibers drawn at 1900 ºC from preform #1. Features of YPO_4_ cubic-shaped nanocrystals are the same as the ones observed at this drawing temperature in preform #3.
